# Supplementary material for: Development of Machine Learning Models for Prediction of Osteoporosis from Clinical Health Examination Data
Source: Int J Environ Res Public Health. 2021 Jul 18;18(14):7635. doi: 10.3390/ijerph18147635 (PMC8305021; doi:10.3390/ijerph18147635)
Supplement: Supplementary file 1 [file ijerph-18-07635-s001.zip › ijerph-1258605-supplementary.pdf]

Supplementary files

Table S1. Domain of the candidate features and the selected features.

	Domain of features	Considered feature	Selected feature
History taking	Personal history	History of smoking	V
		History of alcohol drinking	V
	Medical history	History of Hypertension	
		History of Diabetes mellitus	
	OBGYN history (for Female)	Gravidity	
		Parity	V
		Menopause status	V
		History of HRT	V
Physical examination	Physical characteristics	Age	V
		Height	V
		Weight	V
		Body fat	
		Waist circumference	V
		Buttock circumference	
	Vital signs	Systolic blood pressure	
		Diastolic blood pressure	
		Pulse rate	
Laboratory test	Hematological profile	White blood count	
		Hemoglobin	V
		Platelet	
	Renal function	Blood urea nitrogen	
		Creatinine	V
	Electrolytes	Sodium	
		Potassium	
		Calcium	
		Phosphate	
	Liver function	Total bilirubin	
		Alanine transaminase	V
		Aspartate aminotransferase	
		Alkaline phosphatase	V
	Thyroid function	Thyroid-stimulating hormone	V
		Free thyroxine	
	Lipid profile	Total cholesterol	
		Triglyceride	V
		HDL-C	V
		LDL-C	
	Protein content	Total protein	
		Albumin	V

	Markers for diabetes mellitus	Fasting glucose<br>Postprandial glucose<br>Hemoglobin A1c	
Integrated results		Hypertension<br>Diabetes mellitus	V<br>V

OBGYN: obstetrics and gynecology; HRT: hormone-replacement therapy; HDL-C: high-density lipoprotein cholesterol; LDL-C: low-density lipoprotein cholesterol.

Table S2. Performance of the full models and the smaller models with 6 variables for prediction of osteoporosis in men and women.

	AUROC of full models (95% CI)	AUROC of models with 6 variables (95% CI)	p-value*
Men			
ANN	0.837 (0.805-0.865)	0.779 (0.744-0.812)	0.0682
SVM	0.840 (0.809-0.868)	0.812 (0.779-0.842)	0.2526
RF	0.843 (0.812-0.871)	0.812 (0.779-0.843)	0.2725
KNN	0.821 (0.788-0.851)	0.775 (0.740-0.807)	0.1388
LoR	0.827 (0.794-0.856)	0.782 (0.747-0.814)	0.0153
Women			
ANN	0.781 (0.745-0.814)	0.762 (0.726-0.796)	0.1704
SVM	0.807 (0.773-0.838)	0.783 (0.747-0.816)	0.3643
RF	0.811 (0.777-0.842)	0.785 (0.749-0.817)	0.1778
KNN	0.767 (0.731-0.801)	0.774 (0.738-0.807)	0.8242
LoR	0.772 (0.732-0.806)	0.761 (0.724-0.795)	0.4470

*P-values were calculated with the nonparametric methods to compare two ROC curves proposed by DeLong et al.
ANN: Artificial neural network; SVM: Support vector machine; RF: Random forest; KNN: K-nearest neighbors; LoR: Logistic regression; AUROC: Area under the receiver operating characteristic curve; CI: Confidence interval; ROC curve: receiver operating characteristic curve.
